# Impact of Early Post-Transplantation Diabetes Mellitus and Changes in Diabetic Status on Graft Failure and Mortality in Kidney Transplant Recipients

**DOI:** 10.3389/ti.2026.15476

**Published:** 2026-04-30

**Authors:** Junseok Jeon, Hye Ryoun Jang, Yebin Park, Kyungho Lee, Jung Eun Lee, Kyungdo Han, Wooseong Huh

**Affiliations:** 1 Division of Nephrology, Department of Medicine, Samsung Medical Center, Sungkyunkwan University School of Medicine, Seoul, Republic of Korea; 2 Department of Statistics and Actuarial Science, Soongsil University, Seoul, Republic of Korea

**Keywords:** all-cause mortality, diabetic status, early post-transplantation diabetes mellitus, graft failure, kidney transplantation

## Abstract

Post-transplantation diabetes mellitus (PTDM) is a common complication following kidney transplantation (KT), but the prognostic significance of early PTDM and changes in diabetic status remains uncertain. Using the Korean National Health Insurance Service (NHIS) database, we analyzed 8,486 KT recipients (KTRs) who underwent national health screening from 2009 to 2017. Early PTDM was defined as new-onset diabetes between 3 months and 1 year after KT. Cox regression was used to estimate the risk of graft failure and all-cause mortality. Early PTDM and preexisting DM were present in 12.2% and 28.5% of KTRs, respectively. Early PTDM was significantly associated with mortality (aHR 1.309, 95% CI 1.015–1.689) but not with graft failure. Changes in diabetic status were not significantly associated with graft failure. However, transitioning from non-DM to PTDM (aHR 1.646, 95% CI 1.080–2.510) and having persistent early PTDM (aHR 1.755, 95% CI 1.325–2.377) were associated with increased mortality, whereas regression from early PTDM to non-DM was not. Preexisting DM was associated with increased mortality, regardless of subsequent changes; the risk was relatively lower in those who regressed to non-DM. Changes in diabetic status were found to have a greater impact on outcomes than early PTDM, highlighting the importance of continuous glycemic monitoring and individualized care in KTRs.

## Graphical Abstract

**Figure:**
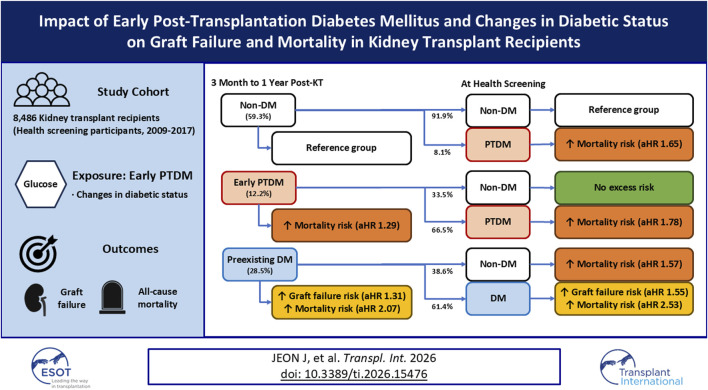

## Introduction

Given its ability to provide a better quality of life and long-term survival compared with dialysis, kidney transplantation (KT) remains the gold standard of renal replacement therapy for eligible patients with end-stage kidney disease [1]. The improvement in the prognosis of KT recipients (KTRs) over the decades highlights the importance of managing long-term complications. Post-transplantation diabetes mellitus (PTDM), defined as newly diagnosed diabetes mellitus (DM) following organ transplantation, is a common complication of KT that occurs in 8%–39% of KTRs with no history of diabetes [2].

Previous studies have revealed an association between PTDM and poor graft or patient outcomes [3–9]. However, some recent studies have found no association between the prognosis of PTDM and KTRs [10–12]. PTDM may serve as an indirect indicator of common factors affecting the overall prognosis, such as the use of high doses of glucocorticoids or calcineurin inhibitors and sarcopenia [13, 14]. Notably, in some studies, PTDM was defined as newly diagnosed DM following KT, regardless of the timing of diagnosis [5, 7, 8]. However, PTDM that develops early after KT may be attributed to the use of immunosuppressants or general health conditions. Furthermore, its impact on patient outcomes differs from that of PTDM that develops later. Thus, the clinical implications of PTDM, particularly early-onset PTDM, remain to be established.

Although attempts have been made to clarify the prognostic impact of PTDM in large cohorts using national health insurance databases, these data contain limited information regarding patient characteristics, such as the glomerular filtration rate and body mass index (BMI) [8, 9]. Therefore, this study aimed to determine the prognostic impact of PTDM among KTRs who participated in a national health screening program, with a focus on the development of early PTDM within 1 year post-KT and the change in diabetic status, to overcome this limitation.

## Materials and Methods

### Data Sources and Study Setting

Data from the Korean National Health Insurance Service (NHIS), which provides mandatory universal coverage to approximately 97% of the Korean population (the Medical Aid Program covers the remaining 3% of the population), were used in this nationwide retrospective cohort study. The NHIS database comprises two databases: an eligibility database (containing information such as age, sex, type and severity of disability, socioeconomic variables, income level, and type of eligibility) and a medical treatment database (containing medical bills submitted by medical service providers). Several epidemiological studies have used data from the Korean NHIS database [15–17].

The NHIS conducts an annual or biennial national health screening program that comprises a standardized self-report questionnaire regarding health behaviors, anthropometric measurements, and laboratory tests. Furthermore, BMI, waist circumference, blood pressure, fasting blood glucose, serum creatinine, triglyceride, and urine protein levels are measured after overnight fasting in accordance with the South Korean Association of Laboratory Quality Control guidelines. To receive reimbursement, medical institutions must adhere to NHIS protocols and undergo certification for quality control procedures [18].

### Ethics Approval

This study was approved by the Institutional Review Board of the Samsung Medical Center (IRB No. 2023-01-006) and adhered to the Declaration of Helsinki. The requirement for obtaining informed consent from the patients was waived owing to the use of anonymized and de-identified data.

### Study Population

**FIGURE 1:**
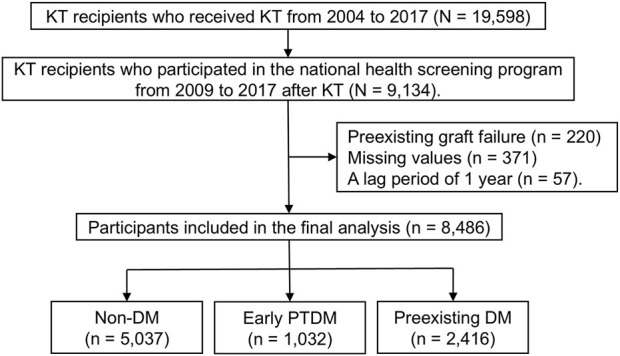
Participant selection process. KT, kidney transplantation.

A total of 19,598 patients who had undergone KT between 2004 and 2017 were identified. A combination of the *International Statistical Classification of Diseases and Related Health Problems, 10th Revision* (ICD-10) code Z94.0 (Kidney transplant status) and a special code for KT (V005) was used to define KT status [19]. The NHIS has registered patients through the Rare and Incurable Disease Registration Program since 2006 to identify patients requiring additional assistance for the management of rare or intractable diseases, including KT status. Patients under this registration are beneficiaries of special medical aid. KTRs who participated in the national health screening program between 2009 and 2017 (N = 9,134) were eligible for inclusion in the present study. Participants with preexisting graft failure (n = 220) and missing values (n = 371) were excluded. Furthermore, a lag period of 1 year was applied to exclude newly diagnosed cases of graft failure or death within 1 year of the national health screening date (n = 57). Thus, the final analysis included 8,486 participants. The participant selection process is shown in Figure 1.

### Exposure: Early PTDM

KTRs were divided into three groups: non-DM, early PTDM, and preexisting DM. Early PTDM was defined as DM newly diagnosed between 3 months and 1 year after KT. Patients who were diagnosed with DM within the first 3 months post-transplant but did not have DM thereafter were classified as non-DM [20]. Preexisting DM was defined as a diagnosis of DM prior to KT. The diagnosis of DM was defined based on the recorded use of at least one oral antidiabetic agent or insulin, with ICD-10 codes E11–E14, or a fasting glucose level of ≥126 mg/dL.

### Covariates

The glomerular filtration rate (eGFR) was determined by assessing the serum creatinine-based eGFR calculated using the Modification of Diet in Renal Disease equation [21]. The dipstick test was used to assess proteinuria, with the detection of traces classified as negative. The presence of at least one claim per year for antihypertensive agents with ICD-10 codes I10–I13 or I15 or a systolic/diastolic blood pressure of ≥140/90 mmHg was defined as hypertension (HTN). The presence of at least one claim per year for antihyperlipidemic agents with ICD-10 code E78 or total cholesterol levels of ≥240 mg/dL was defined as dyslipidemia. A BMI of ≥25 kg/m^2^ was defined as obesity. The ICD-10 codes F32 or F33 indicated a diagnosis of depression. Smoking status was categorized as never, former, or current smoker. Alcohol consumption was categorized as none, moderate (<30 g/day), and heavy (≥30 g/day). Regular exercise was defined as engaging in moderate physical activity for >30 min ≥5 times per week or engaging in strenuous physical activity for >20 min ≥3 times per week. The low-income group comprised those in the bottom 25% of earners. The requirement for rituximab, thymoglobulin, and intravenous glucocorticoids for >3 days; intravenous immune globulin; or plasmapheresis following readmission after KT was defined as acute rejection therapy.

### Outcomes and Follow-Up

The primary outcome measures were graft failure and all-cause mortality. Graft failure, defined as receiving dialysis at least 3 months post-KT (hemodialysis > at least 25 times or peritoneal dialysis at least 3 months), was assessed using death-censored graft failure. The follow-up period commenced on the date of the national health screening examination and ended at the occurrence of the outcome on 31 December 2021.

### Subgroup Analysis

Subgroup analyses were conducted based on age (<40, 40–49, 50–59, 60–69, and ≥70 years old), sex, low income, smoking status (never or former vs. current), alcohol consumption, regular exercise, hypertension, dyslipidemia, depression, proteinuria, hospitalization within 1 year post-KT, and acute rejection therapy within 1 year post-KT.

### Sensitivity Analysis

The majority of previous studies defined PTDM to include DM diagnosed within the first 3 months after KT. For comparison, we performed an additional sensitivity analysis using this conventional definition, classifying all patients diagnosed with DM within 1 year post-transplant as having PTDM.

### Statistical Analysis

Categorical and continuous variables are presented as numbers (percentages) and mean ± standard deviation or median (interquartile range), respectively. Intergroup comparisons were conducted using a t-test (for continuous variables) and a Chi-square test (for categorical variables). The incidence rate (IR) was presented per 1,000 person-years. Cumulative graft failure and all-cause mortality were expressed using Kaplan–Meier curves and compared using the log-rank test. Hazard ratios (HR) with 95% confidence intervals (CI) were estimated using Cox proportional hazards regression analysis to estimate the risk of graft failure and all-cause mortality. Multivariable analysis was conducted for the following variables: sex, age, low income, smoking status, alcohol consumption, regular exercise, HTN, dyslipidemia, depression, BMI, eGFR, the time from KT to screening, proteinuria, induction therapy, and acute rejection within 1 year post-KT. All statistical analyses were conducted using SAS 9.4 (SAS Institute, Cary, North Carolina, USA). A two-sided *P*-value of <0.05 was considered statistically significant.

## Results

### Characteristics of the Study Population

Table 1 presents the characteristics of KTRs at the time of participating in the national health screening program. Of the 8,486 KTRs, 1,032 (12.2%) and 2,416 (28.5%) KTRs had early PTDM and preexisting DM, respectively. The mean interval between KT and health screening was 3.04 ± 2.41 years. The mean age was 50.1 ± 10.4 years, and 5,062 (59.63%) of the KTRs were men. Patients in the preexisting DM group were the oldest and most likely to be men, followed by those in the early PTDM group. The BMI and waist circumference were highest in the preexisting DM group, followed by the early PTDM group. Systolic blood pressure was lowest in the non-DM group and similar between the early PTDM and preexisting DM groups; however, diastolic blood pressure and the proportion of HTN were highest in the early PTDM group. The eGFR was similar across the three groups, whereas the early PTDM group had the highest proportion of proteinuria. Total HDL-, and LDL-cholesterol levels were highest in the non-DM group; however, triglyceride levels and the proportion of dyslipidemia were the highest in the early PTDM group. The proportion of KTRs with low income was the lowest in the non-DM group. The median (interquartile range) follow-up durations for graft failure and all-cause mortality were 6.12 (4.22–8.40) years and 6.00 (4.06–8.14) years, respectively.

**TABLE 1:** Baseline characteristics of KT recipients.

Variables	All	Non-DM	Early PTDM	Preexisting DM	P- value
	(N = 8,486)	(n = 5,037)	(n = 1,032)	(n = 2,416)	
Age, y	50.1 ± 10.4	47.68 ± 10.46	53.01 ± 9.27	53.74 ± 9.32	<0.0001
<40	1,263 (14.88)	1,022 (20.29)	75 (7.27)	166 (6.87)	<0.0001
40–49	2,506 (29.53)	1,693 (33.60)	251 (24.32)	562 (23.26)	​
50–59	3,080 (36.3)	1,646 (32.67)	449 (43.51)	985 (40.77)	​
60–69	1,484 (17.49)	631 (12.52)	230 (22.29)	623 (25.79)	​
≥70	153 (1.80)	46 (0.91)	27 (2.62)	80 (3.31)	​
Male sex	5,062 (59.65)	2,841 (56.39)	634 (61.43)	1,587 (65.69)	<0.0001
BMI, kg/m^2^	22.90 ± 3.28	22.66 ± 3.22	23.11 ± 3.17	23.32 ± 3.42	<0.0001
Waist circumference, cm	79.97 ± 9.59	78.66 ± 9.24	81.06 ± 9.2	82.22 ± 9.99	<0.0001
Systolic BP, mmHg	125.48 ± 14.91	124.89 ± 14.23	126.19 ± 14.98	126.4 ± 16.15	<0.0001
Diastolic BP, mmHg	78.12 ± 10.22	78.84 ± 10.02	79.11 ± 10.41	76.2 ± 10.32	<0.0001
Hypertension	5,651 (66.59)	3,203 (63.58)	743 (72.00)	1,705 (70.57)	<0.0001
Dyslipidemia	4,880 (57.51)	2,767 (54.92)	667 (64.63)	1,446 (59.85)	<0.0001
Depression	504 (5.94)	240 (4.76)	71 (6.88)	193 (7.99)	<0.0001
eGFR, mL/min/1.73 m^2^	65.49 ± 42.96	65.73 ± 42.94	63.41 ± 41.01	65.89 ± 43.78	0.248
Proteinuria, positive	858 (10.11)	471 (9.35)	126 (12.21)	261 (10.8)	0.0087
Fasting glucose, mg/dL	107.89 ± 35.29	97.68 ± 18.05	117.07 ± 35.40	125.26 ± 51.04	<0.0001
Total cholesterol, mg/dL	183.38 ± 36.08	186.21 ± 35.25	181.72 ± 34.97	178.17 ± 37.63	<0.0001
HDL-C, mg/dL	58.76 ± 20.87	60.71 ± 23.38	55.69 ± 15.33	56.00 ± 16.47	<0.0001
LDL-C, mg/dL	99.07 ± 31.02	100.51 ± 30.92	97.31 ± 30.29	96.83 ± 31.38	<0.0001
Triglyceride^a^, mg/dL	115 (114–117)	114 (113–116)	129 (126–133)	112 (110–114)	<0.0001
Smoking status	​	​	​	​	<0.0001
Never smoker	5,208 (61.37)	3,227 (64.05)	627 (60.76)	1,354 (56.04)	​
Former smoker	2,731 (32.18)	1,512 (30.01)	337 (32.66)	882 (36.51)	​
Current smoker	547 (6.45)	299 (5.93)	68 (6.59)	180 (7.45)	​
Alcohol consumption	​	​	​	​	0.001
None	7,291 (85.92)	4,277 (84.89)	888 (86.05)	2,126 (88.00)	​
Moderate	1,127 (13.28)	725 (14.39)	137 (13.28)	265 (10.97)	​
Heavy	68 (0.8)	36 (0.71)	7 (0.68)	25 (1.03)	​
Regular exercise	2,128 (25.08)	1,201 (23.84)	270 (26.16)	657 (27.19)	0.0052
Low income (<20%)	2,434 (28.68)	1,383 (27.45)	318 (30.81)	733 (30.34)	0.0097
Time from KT to screening, y	3.04 ± 2.41	3.25 ± 2.52	3.10 ± 2.36	2.59 ± 2.13	<0.0001

Categorical variables and continuous variables are presented as numbers (percentages) and as mean ± standard deviation.

^a^ Triglyceride values are shown as a geometric mean (95% confidence interval).

BMI, body mass index; BP, blood pressure; DM, diabetes mellitus; eGFR, estimated glomerular filtration rate; HDL-C, high-density lipoprotein-cholesterol; KT, kidney transplantation; KTRs, recipients of KT; LDL-C, low-density lipoprotein-cholesterol; PTDM, post-transplantation diabetes mellitus.

### Treatment History During the First Year Post-KT

Table 2 presents the treatment history during the first year post-KT. The proportion of KTRs who did not receive induction therapy was the highest in the non-DM group (14.4%), followed by the early PTDM group (10.3%) and the preexisting DM group (6.3%). The proportion of KTRs who underwent thymoglobulin induction in the preexisting DM group (11.0%) was significantly higher than that in the non-DM (6.8%) and early PTDM (6.6%) groups. Acute rejection therapy commenced within 1 year post-KT in 7.34% of KTRs. The incidence of acute rejection was the highest in the early PTDM group (10.9%), followed by the preexisting DM (8.3%) and non-DM (6.2%) groups.

**TABLE 2:** Treatment history during the first year post-KT.

Variables	All	Non-DM	Early PTDM	Preexisting DM	P value
Induction therapy	​	​	​	​	<0.0001
None	983 (11.58)	724 (14.37)	106 (10.27)	153 (6.33)	​
Thymoglobulin	675 (7.95)	340 (6.75)	69 (6.69)	266 (11.01)	​
Basiliximab	6,617 (77.98)	3,858 (76.58)	825 (79.94)	1,934 (80.05)	​
Both	211 (2.49)	116 (2.30)	32 (3.1)	63 (2.61)	​
Acute rejection within 1 year	623 (7.34)	311 (6.17)	112 (10.85)	200 (8.28)	<0.0001
OPD visits within 1 year, n	25.26 ± 14.04	23.01 ± 12.41	25.58 ± 12.55	29.82 ± 16.51	<0.0001
<10	60 (0.71)	54 (1.07)	1 (0.10)	5 (0.21)	<0.0001
10–19	3,296 (38.84)	2,343 (46.51)	362 (35.08)	591 (24.46)	​
20–29	2,963 (34.92)	1,671 (33.17)	391 (37.89)	901 (37.29)	​
30–39	1,222 (14.4)	583 (11.57)	175 (16.96)	464 (19.21)	​
≥40	945 (11.14)	387 (7.68)	103 (9.98)	455 (18.83)	​
Hospitalizations within 1 year, n	​	​	​	​	<0.0001
0	1,923 (22.66)	1,344 (26.68)	181 (17.54)	398 (16.47)	​
1	2,846 (33.54)	1,768 (35.09)	316 (30.62)	762 (31.54)	​
≥2	3,717 (43.80)	1,926 (38.23)	535 (51.84)	1,256 (51.99)	​

Categorical variables are presented as numbers (percentages).

Abbreviations: DM, diabetes mellitus; KT, kidney transplantation; PTDM, post-transplantation diabetes mellitus; OPD, outpatient department.

The mean number of outpatient department visits within 1 year post-KT was the highest in the preexisting DM group (29.8 ± 16.5), followed by the early PTDM (25.6 ± 12.6) and non-DM (23.0 ± 12.4) groups. Multiple hospitalizations (≥2 times) within 1 year post-KT were similar in the early PTDM (51.8%) and preexisting DM (52.0%) groups, followed by the non-DM (38.2%) group.

### Risk of Graft Failure and All-Cause Mortality According to Early PTDM within 1 Year Post-KT

**FIGURE 2:**
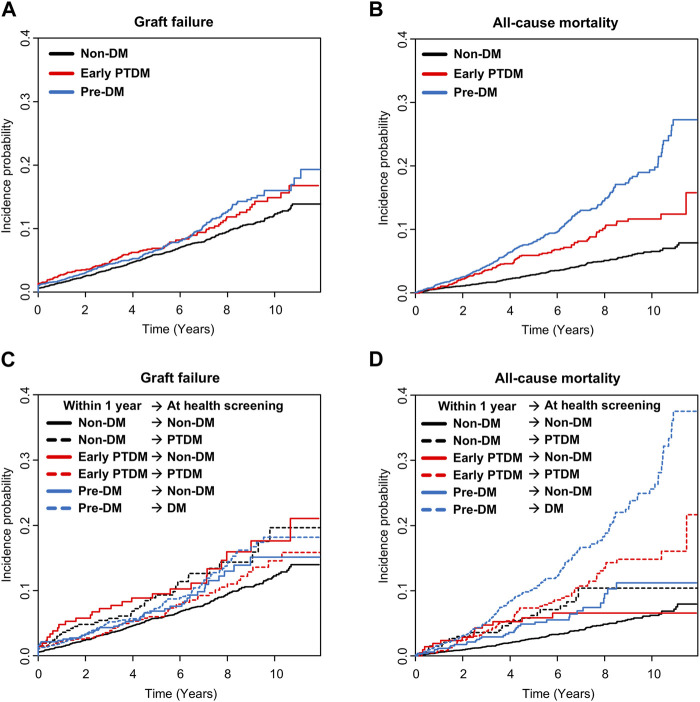
Cumulative incidence of graft failure and all-cause mortality according to diabetic status within 1 year post-KT and changes in diabetic status in KT recipients who underwent health screening following KT. **(A)** Graft failure according to the diabetic status within 1 year post-KT, **(B)** all-cause mortality according to diabetic status within 1 year post-KT, **(C)** graft failure according to the changes in diabetic status, and **(D)** all-cause mortality according to the changes in diabetic status. DM, diabetes mellitus; KT, kidney transplantation; PTDM, post-transplantation diabetes mellitus.

Compared with the non-DM group, the early PTDM group exhibited no association with the risk of graft failure but a significant association with the risk of all-cause mortality (adjusted hazard ratio [aHR] 1.044, 95% CI 0.833–1.309 for graft failure and 1.309, 95% CI 1.015–1.689 for all-cause mortality) (Table 3). Figures 2A,B present the cumulative incidences of all-cause mortality and graft failure according to the diabetic status 1 year post-KT. Compared with that in the non-DM group, an increased risk of graft failure and all-cause mortality was observed in the preexisting DM group (Table 3).

**TABLE 3:** Risk of graft failure and mortality according to diabetic status within 1 year post-KT.

Outcome	Diabetic status	IR^a^	Hazard ratio (95% confidence interval)			
			Model 1	Model 2	Model 3	Model 4
Graft failure	Non-DM	12.49	1 (Ref.)	1 (Ref.)	1 (Ref.)	1 (Ref.)
	Early PTDM	15.49	1.240 (0.995, 1.545)	1.359 (1.086, 1.699)	1.284 (1.026, 1.606)	1.044 (0.833, 1.309)
	Preexisting DM	15.57	1.266 (1.071, 1.496)	1.400 (1.176, 1.666)	1.344 (1.129, 1.600)	1.287 (1.079, 1.535)
All-cause mortality	Non-DM	6.33	1 (Ref.)	1 (Ref.)	1 (Ref.)	1 (Ref.)
	Early PTDM	12.62	2.003 (1.560, 2.573)	1.424 (1.107, 1.833)	1.422 (1.105, 1.830)	1.309 (1.015, 1.689)
	Preexisting DM	19.29	3.169 (2.648, 3.792)	2.064 (1.715, 2.483)	2.005 (1.665, 2.414)	2.065 (1.712, 2.491)

Model 1 was unadjusted; Model 2 was adjusted for sex and age; Model 3 was further adjusted for low income, smoking status, alcohol consumption, regular exercise, and hypertension, dyslipidemia, and depression; and Model 4 was additionally adjusted for body mass index, estimated glomerular filtration rate, proteinuria, time from kidney transplantation to health screening, induction therapy, and acute rejection within 1 year.

^a^ Incidence rates are expressed per 1,000 person-years.

DM, diabetes mellitus; IR, incidence rate; KT, kidney transplantation; PTDM, post-transplantation diabetes mellitus.

### Risk of Graft Failure and All-Cause Mortality According to Changes in Diabetic Status

The risk of graft failure and all-cause mortality was evaluated according to changes in diabetic status from 1 year post-KT to the time of health screening (Table 4). An analysis was conducted after excluding KTRs who underwent health screening within 1 year post-KT to distinguish between early diabetic status and the changes in diabetic status. While 8.1% of non-DM patients progressed to PTDM, 33.5% of those with early PTDM and 38.6% of those with preexisting DM regressed to a non-DM status. KTRs who remained non-diabetic from KT until health screening served as the reference group.

**TABLE 4:** Risk of graft failure and mortality according to changes in diabetic status.

Outcome	Changes in diabetic status		N	IR^a^	Hazard ratio (95% confidence interval)			
	With 1 year after KT	At health screening			Model 1	Model 2	Model 3	Model 4
Graft failure	Non-DM	Non-DM	3,807	12.24	1 (Ref.)	1 (Ref.)	1 (Ref.)	1 (Ref.)
	Non-DM	PTDM	336	19.77	1.625 (1.164, 2.268)	1.707 (1.221, 2.385)	1.506 (1.074, 2.111)	1.314 (0.934, 1.848)
	Early PTDM	Non-DM	211	20.37	1.655 (1.116, 2.454)	1.803 (1.215, 2.678)	1.695 (1.141, 2.518)	1.221 (0.818, 1.824)
	Early PTDM	PTDM	629	14.38	1.175 (0.883, 1.563)	1.349 (1.009, 1.804)	1.236 (0.923, 1.654)	1.034 (0.770, 1.389)
	Pre-DM	Non-DM	514	15.47	1.283 (0.940, 1.750)	1.350 (0.989, 1.842)	1.302 (0.953, 1.779)	1.298 (0.948, 1.779)
	Pre-DM	DM	1,332	17.10	1.422 (1.154, 1.753)	1.701 (1.359, 2.128)	1.608 (1.284, 2.013)	1.545 (1.231, 1.940)
All-cause mortality	Non-DM	Non-DM	3,807	6.02	1 (Ref.)	1 (Ref.)	1 (Ref.)	1 (Ref.)
	Non-DM	PTDM	336	12.31	2.076 (1.370, 3.144)	1.891 (1.248, 2.866)	1.809 (1.191, 2.749)	1.646 (1.080, 2.510)
	Early PTDM	Non-DM	211	9.00	1.478 (0.839, 2.602)	1.127 (0.639, 1.988)	1.099 (0.623, 1.938)	0.864 (0.488, 1.530)
	Early PTDM	PTDM	629	16.24	2.719 (2.041, 3.621)	1.899 (1.422, 2.535)	1.886 (1.412, 2.521)	1.775 (1.325, 2.377)
	Pre-DM	Non-DM	514	10.86	1.864 (1.286, 2.703)	1.629 (1.123, 2.363)	1.556 (1.073, 2.257)	1.566 (1.077, 2.275)
	Pre-DM	DM	1,332	25.67	4.455 (3.607, 5.504)	2.577 (2.066, 3.214)	2.465 (1.973, 3.080)	2.532 (2.022, 3.169)

Model 1 was unadjusted; Model 2 was adjusted for sex and age; Model 3 was further adjusted for low income, smoking status, alcohol consumption, regular exercise, and hypertension, dyslipidemia, and depression; and Model 4 was additionally adjusted for body mass index, estimated glomerular filtration rate, proteinuria, time from kidney transplantation to health screening, induction therapy, and acute rejection within 1 year.

^a^ Incidence rates are expressed per 1,000 person-years.

DM, diabetes mellitus; IR, incidence rate; KT, kidney transplantation; PTDM, post-transplantation diabetes mellitus.

KTRs who transitioned from non-DM to PTDM or from early PTDM to non-DM showed a significantly higher risk of graft failure in the univariable analysis. However, in the multivariable model, these transition groups did not exhibit a statistically significant increase in risk of graft failure, although transition from non-DM to PTDM tended to be associated with an increased risk (non-DM → PTDM: aHR 1.314, 95% CI 0.934–1.848; early PTDM → non-DM: aHR 1.221, 95% CI 0.818–1.824). Patients who remained in the PTDM status (early PTDM → PTDM) did not show a significantly elevated risk of graft failure. Among KTRs with preexisting DM, those who remained diabetic had a significantly higher risk of graft failure compared with those who remained non-diabetic throughout (aHR 1.545, 95% CI 1.231–1.940), whereas those who transitioned to non-DM did not show a significantly increased risk (aHR 1.298, 95% CI 0.948–1.779). Figure 2C presents the cumulative incidence of graft failure according to the changes in diabetic status.

KTRs who transitioned from non-DM to PTDM, along with those with persistent early PTDM, exhibited a significantly higher risk of all-cause mortality (non-DM → PTDM: aHR 1.646, 95% CI 1.080–2.510; early PTDM → PTDM: aHR 1.755, 95% CI 1.325–2.377) (Table 4). In contrast, KTRs who transitioned from early PTDM to non-DM showed no significant association with all-cause mortality. Among those with preexisting DM, the risk of all-cause mortality was increased regardless of subsequent changes in diabetic status, although those who transitioned from preexisting DM to non-DM exhibited a relatively lower HR for all-cause mortality compared with those who remained diabetic. Figure 2D presents the cumulative incidence of all-cause mortality according to the changes in diabetic status.

A sensitivity analysis conducted after excluding KTRs who underwent health screening within 2 years post-KT yielded comparable results, except that transitioning from non-DM to PTDM was significantly associated with graft failure (aHR 1.473, 95% CI 1.025–2.118) (Supplementary Table S1).

### Subgroup Analysis

**FIGURE 3:**
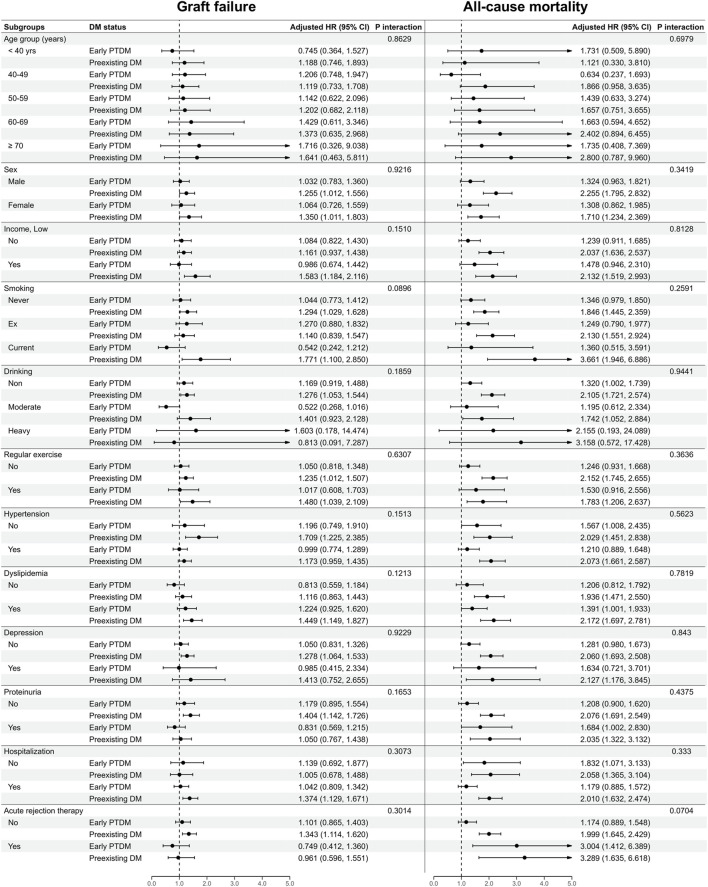
Subgroup analysis for graft failure and all-cause mortality according to diabetic status within 1 year post-KT. CI, confidence interval; DM, diabetes mellitus; HR, hazard ratio; KT, kidney transplantation; PTDM, post-transplantation diabetes mellitus.

Figure 3 and Supplementary Tables S2, S3 present the results of the subgroup analyses. The relationship between early PTDM and graft failure or all-cause mortality exhibited no significant interaction with respect to age, sex, income, smoking status, alcohol consumption, regular exercise, HTN, dyslipidemia, proteinuria, hospitalization, or acute rejection therapy.

### Outcomes With an Early PTDM Definition, Including the First 3 Months Post-KT

When the first 3 months post-KT were included in the definition of early PTDM, the number of early PTDM cases increased by 921, from 1,032 to 1,953. Under this definition, the HRs of early PTDM were attenuated and were not significantly associated with the risk of either graft failure or all-cause mortality (Supplementary Table S4). In the analysis of changes in diabetic status, HRs were amplified in both directions; that is, higher HRs became higher, and lower HRs became lower. Consequently, KTRs who transitioned from non-DM to PTDM showed a significantly increased risk of graft failure and all-cause mortality (non-DM → PTDM: aHR 1.578, 95% Cl 1.074–2.320 for graft failure and aHR 1.784, 95% Cl 1.100–2.893 for all-cause mortality). Notably, patients who transitioned from early PTDM to non-DM demonstrated an even lower risk of all-cause mortality compared with those who consistently remained non-DM (early PTDM → non-DM: aHR 0.678, 95% Cl 0.464–0.992) (Supplementary Table S5).

### Analysis of all KTRs Irrespective of Health Screening Participation

We further analyzed all 24,524 KTRs who underwent KT between 2004 and 2020, regardless of their participation in the national health screening program, after excluding those with missing values (n = 536) and those who died <3 months post-KT (n = 309). Supplementary Table S6 summarizes the characteristics of all KTRs who underwent KT between 2004 and 2020; the characteristics were similar to those of KTRs who participated in the health screening. Treatment history patterns of all KTRs according to the diabetic status were also similar to those of KTRs who participated in the national health screening program. However, the proportions of KTRs who underwent thymoglobulin induction (13.51% in all KTRs vs. 7.95% in KTRs who participated in the national health screening program) and acute rejection therapy within 1 year (10.69% in all KTRs vs. 7.34% in KTRs who participated in the health screening) tended to be higher. Furthermore, the mean number of hospitalizations within 1 year was higher among all KTRs compared with that among those who participated in the national health screening program (2.05 in all KTRs vs. 1.68 in KTRs who participated in the national health screening program).

Compared with non-DM, early PTDM was associated with an increased risk of graft failure and all-cause mortality in all KTRs who underwent KT between 2004 and 2020 (aHR 1.524, 95% CI 1.344–1.729 for graft failure and 1.339, 95% CI 1.153–1.535 for all-cause mortality) (Table 5).

**TABLE 5:** Risk of graft failure and mortality by diabetic status within 1 year of KT in all KTRs who received a KT from 2004 to 2020, regardless of health screening participation.

Outcome	Diabetic status	N	IR^a^	Hazard ratio (95% confidence interval)		
				Model 1	Model 2	Model 3
Graft failure	Non-DM	13,546	11.39	1 (Ref.)	1 (Ref.)	1 (Ref.)
	Early PTDM	2,742	17.32	1.525 (1.349, 1.725)	1.708 (1.506, 1.936)	1.524 (1.344, 1.729)
	Preexisting DM	8,954	15.24	1.385 (1.264, 1.519)	1.579 (1.433, 1.741)	1.462 (1.324, 1.614)
All-cause mortality	Non-DM	13,546	6.22	1 (Ref.)	1 (Ref.)	1 (Ref.)
	Early PTDM	2,742	13.24	2.142 (1.861, 2.465)	1.474 (1.279, 1.700)	1.339 (1.153, 1.535)
	Preexisting DM	8,954	20.03	3.432 (3.113, 3.783)	2.161 (1.952, 2.392)	2.108 (1.900, 2.338)

Model 1 was unadjusted; Model 2 was adjusted for sex and age; Model 3 was further adjusted for low income, hypertension, dyslipidemia, induction therapy, and acute rejection within 1 year.

^a^ Incidence rates are expressed per 1,000 person-years.

DM, diabetes mellitus; IR, incidence rate; KT, kidney transplantation; PTDM, post-transplantation diabetes mellitus.

## Discussion

This nationwide study in the Republic of Korea demonstrated that early PTDM, defined as the onset of DM between 3 months and 1 year post-KT, was not associated with graft failure but was significantly associated with a higher risk of all-cause mortality in KTRs who participated in a national health screening program post-KT. However, early PTDM was associated with a higher risk of graft failure in the overall KTR population. Changes in diabetic status from within to beyond 1 year after KT may serve as a more significant prognostic factor than early PTDM alone. Notably, KTRs with early PTDM who no longer had diabetes later exhibited no increased risk of mortality, whereas those who transitioned from non-DM to PTDM showed a higher risk of mortality. Notably, over one-third of patients with preexisting DM regressed to non-DM status. Nevertheless, preexisting DM was associated with a higher risk of mortality even among those who transitioned to non-DM, whereas graft failure risk was not elevated in this group.

Few studies have explored the association between early PTDM within 1 year post-KT and outcomes, and their results have been conflicting [3, 11]. One study reported that PTDM developing within 1 year post-KT was not associated with adverse outcomes, whereas PTDM developing after 1 year was associated with adverse outcomes [11]. This suggests that the prognostic impact of PTDM may vary depending on the timing of the onset, consistent with our findings. The timing of PTDM diagnosis is closely related to how PTDM is defined. The majority of studies included early post-transplant hyperglycemia as PTDM, and only a few excluded the first 1–3 months post-KT [9, 22, 23]. A recent consensus recommends performing the first diagnostic glucose tolerance test at 10–13 weeks post-KT, classifying earlier hyperglycemia as persistent hyperglycemia rather than PTDM [20]. Our study addressed this issue by defining early PTDM as new-onset diabetes occurring between 3 months and 1 year post-KT. When patients with hyperglycemia within 3 months were classified as non-DM, the HRs of PTDM for outcomes increased, suggesting that these patients may have a lower risk of complications. These findings support the current consensus recommending exclusion of hyperglycemia within 3 months of a diagnosis of PTDM.

The present study investigated the prognostic impact of the changes in diabetic status in the first year post-KT and beyond. Regression from early PTDM to non-DM was not associated with an increased risk of graft failure or mortality, whereas new-onset PTDM and persistent PTDM beyond 1 year post-KT were linked to worse outcomes. Preexisting DM conferred a persistent risk of mortality even after regression to non-DM, although the HR was lower than that of those with persistent DM. Some degree of hyperglycemia may be an appropriate compensatory response to acute stress conditions such as the early post-KT state. Hyperglycemia may develop as an appropriate response to glucose utilization by tissues in the presence of insulin resistance under acute stressful conditions such as sepsis [24]. In addition, early PTDM may also lead to more frequent medical visits and multidisciplinary care during the early post-transplantation period, thereby facilitating more optimal management. Few studies have evaluated the impact of changes in diabetic status after KT, and their findings have been conflicting [4, 25]. Transient post-KT hyperglycemia (corresponding to early PTDM to non-DM in the present study) was not associated with adverse outcomes in a previous study [25]. In contrast, transient PTDM was associated with poorer patient and graft outcomes than sustained PTDM in another study, irrespective of its onset [4]. Our findings suggest that persistent DM or late-onset PTDM has a greater impact on outcomes than early post-transplant diabetic status. Our study revealed a more comprehensive picture of the dynamic nature and important clinical impact of changes in diabetic status on long-term outcomes post-KT. These findings underscore the importance of monitoring the changes in diabetic status over time after KT and the role of multidisciplinary care in improving the overall outcomes of KTRs with early PTDM or preexisting DM.

Predisposing factors for PTDM in KTRs include the use of glucocorticoids and calcineurin inhibitors, along with viral infections, such as cytomegalovirus [13, 26–28]. Thus, KTRs with high immunological risk or acute rejection who require more intense immunosuppressants are at a higher risk for PTDM and poor outcomes. Glucocorticoids and immunosuppressants can induce sarcopenia [29, 30]. Sarcopenia, which is associated with an increased risk of DM, is also associated with an increased risk of graft failure and mortality among KTRs [31–33]. In contrast, hyperglycemia after KT itself can promote oxidative stress and subsequent endothelial dysfunction, thereby accelerating allograft dysfunction and overall mortality [34]. Determining whether early PTDM is an independent prognostic factor or a marker of poor general status is therefore challenging. KTRs in poor condition after KT were unlikely to participate in the national health screening program. Indeed, the rates of thymoglobulin induction, acute rejection therapy, and hospitalization within 1 year post-KT observed among all KTRs tended to be higher than among national health screening program participants. This selection bias may explain why early PTDM was associated with graft failure among all KTRs but not among national health screening program participants among KTRs. Therefore, our findings might support the importance of actively managing PTDM in KTRs with a higher immunologic risk or poorer general conditions.

The present study has some limitations. First, the retrospective design and the limitations of the NHIS database hindered the capture of more detailed information, such as HLA matching status, donor information, and cause of death. Consequently, the possibility of unmeasured confounders that affected DM status and the associated outcomes cannot be excluded. Second, rather than using HbA1c levels, blood glucose levels, or glucose tolerance test results, the operational definition of DM status was based on diagnostic codes and antidiabetic medication use. However, this operational definition of DM using health insurance claims data has been soundly validated as a reliable tool in a recent study of the Korean population [35]. Third, the KTRs who participated in the national health screening program may be subject to selection bias, as they likely represent only relatively healthy or health-conscious KTRs. However, the relationship between early PTDM and clinical outcomes was assessed more accurately by excluding KTRs with extremely poor post-KT health status or poor compliance. This is a major strength of the present study.

In conclusion, early PTDM was associated with an elevated risk of mortality, while the development of PTDM beyond 1 year was associated with poor outcomes, and the resolution of early PTDM was associated with favorable outcomes. These findings underscore the importance of active surveillance, early intervention, and personalized management strategies for PTDM after KT to improve long-term patient and graft outcomes.

## Data Availability

The datasets presented in this article are not readily available because they are owned by the Korea NHIS. Requests to access the datasets should be directed to https://nhiss.nhis.or.kr/bd/ab/bdaba000eng.do.
