# Supplementary material for: Impact of Early Post-Transplantation Diabetes Mellitus and Changes in Diabetic Status on Graft Failure and Mortality in Kidney Transplant Recipients
Source: Transpl Int. 2026 Apr 30;39:15476. doi: 10.3389/ti.2026.15476 (PMC13171467; doi:10.3389/ti.2026.15476)
Supplement: Supplementary file 1 [file DataSheet1.pdf]

Supplementary Material

Supplement to: Impact of early post-transplantation diabetes mellitus and changes in diabetic status on graft failure and mortality in kidney transplant recipients

Junseok Jeon et al.

Supplementary Table S1. Risk of graft failure and mortality according to the changes in diabetic status excluding national health screening program participation within 2 years post-KT

Supplementary Table S2. Subgroup analysis of graft failure according to diabetic status within one year post-KT

Supplementary Table S3. Subgroup analysis of all-cause mortality according to diabetic status within one year post-KT

Supplementary Table S4. Risk of graft failure and mortality according to diabetic status within one year post-KT: early PTDM defined as onset within the first year after KT

Supplementary Table S5. Risk of graft failure and mortality according to the changes in diabetic status: early PTDM defined as onset within the first year after KT

Supplementary Table S6. Baseline characteristics of all KT recipients who received KT from 2004 to 2020 regardless of national health screening program participation

Supplementary Table S1. Risk of graft failure and mortality according to the changes in diabetic status excluding national health screening progression participation within 2 years post-KT

	diabetic status		N	IR[a]	Hazard ratio (95% confidence interval)			
	With 1 year after KT	At health screening			Model 1	Model 2	Model 3	Model 4
**Graft failure**	**Non-DM**	**Non-DM**	2,714	12.79	1 (Ref.)	1 (Ref.)	1 (Ref.)	1 (Ref.)
	**Non-DM**	**PTDM**	263	23.76	1.863 (1.310, 2.648)	1.947 (1.368, 2.773)	1.716 (1.200, 2.454)	1.473 (1.025, 2.118)
	**Early PTDM**	**Non-DM**	185	18.46	1.435 (0.926, 2.224)	1.576 (1.015, 2.447)	1.504 (0.968, 2.337)	1.147 (0.734, 1.793)
	**Early PTDM**	**PTDM**	417	16.77	1.311 (0.946, 1.817)	1.522 (1.091, 2.124)	1.379 (0.987, 1.926)	1.234 (0.878, 1.733)
	**Pre-DM**	**Non-DM**	344	16.48	1.305 (0.905, 1.881)	1.371 (0.950, 1.977)	1.354 (0.938, 1.954)	1.288 (0.889, 1.866)
	**Pre-DM**	**DM**	816	21.31	1.693 (1.332, 2.151)	2.054 (1.588, 2.656)	1.951 (1.508, 2.525)	1.874 (1.442, 2.434)
**All-cause mortality**	**Non-DM**	**Non-DM**	2,714	6.41	1 (Ref.)	1 (Ref.)	1 (Ref.)	1 (Ref.)
	**Non-DM**	**PTDM**	263	13.99	2.220 (1.420, 3.469)	2.000 (1.279, 3.127)	1.848 (1.177, 2.902)	1.682 (1.066, 2.652)
	**Early PTDM**	**Non-DM**	185	7.73	1.191 (0.625, 2.271)	0.884 (0.463, 1.689)	0.863 (0.451, 1.649)	0.680 (0.354, 1.308)
	**Early PTDM**	**PTDM**	417	19.41	3.067 (2.219, 4.240)	2.065 (1.488, 2.867)	2.009 (1.445, 2.793)	1.876 (1.346, 2.614)
	**Pre-DM**	**Non-DM**	344	11.86	1.915 (1.244, 2.949)	1.666 (1.082, 2.567)	1.613 (1.047, 2.486)	1.597 (1.033, 2.467)
	**Pre-DM**	**DM**	816	26.98	4.390 (3.419, 5.638)	2.503 (1.925, 3.254)	2.382 (1.827, 3.105)	2.372 (1.814, 3.102)

Model 1 was unadjusted; Model 2 was adjusted for sex and age; Model 3 was further adjusted for low income, smoking status, alcohol consumption, regular exercise; and Model 4 was additionally adjusted for hypertension, dyslipidemia, depression, body mass index, estimated glomerular filtration rate, proteinuria, time from kidney transplantation to health screening, induction therapy, and acute rejection within one year.

[a]Incidence rates are expressed as per 1000 person-year

DM, diabetes mellitus; IR, incidence rate; KT, kidney transplantation; PTDM, post-transplantion diabetes mellitus

Supplementary Table S2. Subgroup analysis of graft failure according to diabetic status within one year post-KT.

Subgroup	diabetic status	N	IR	Unadjusted HR (95% CI)	P for interaction	Adjusted HR (95% CI)	P for interaction
**Age group (years)**					0.8397		0.2183
**<40**	**Non-DM**	1022	18.11	1 (Ref.)		1 (Ref.)	
	**Early PTDM**	75	15.32	0.837 (0.409,1.712)		0.531 (0.258, 1.092)	
	**Pre-DM**	166	21.52	1.204 (0.758, 1.913)		0.813 (0.507, 1.305)	
**40–49**	**Non-DM**	1693	12.97	1 (Ref.)		1 (Ref.)	
	**Early PTDM**	251	19.75	1.520 (1.036, 2.230)		1.294 (0.879, 1.906)	
	**Pre-DM**	562	16.60	1.294 (0.946, 1.771)		1.285 (0.935, 1.766)	
**50–59**	**Non-DM**	1646	9.81	1 (Ref.)		1 (Ref.)	
	**Early PTDM**	449	13.80	1.404 (0.972, 2.028)		1.011 (0.697, 1.466)	
	**Pre-DM**	985	14.80	1.525 (1.145, 2.031)		1.465 (1.097, 1.957)	
**60–69**	**Non-DM**	631	8.96	1 (Ref.)		1 (Ref.)	
	**Early PTDM**	230	13.96	1.561 (0.879, 2.772)		1.300 (0.730, 2.315)	
	**Pre-DM**	623	14.22	1.608 (1.028, 2.515)		1.420 (0.905, 2.226)	
**≥70**	**Non-DM**	46	4.31	1 (Ref.)		1(Ref.)	
	**Early PTDM**	27	14.79	3.426 (0.311, 37.697)		4.776 (0.432, 52.823)	
	**Pre-DM**	80	14.49	3.343 (0.392, 28.542)		3.837 (0.448, 32.888)	
**Sex**					0.4377		0.9216
**Male**	**Non-DM**	2841	14.08	1 (Ref.)		1(Ref.)	
	**Early PTDM**	634	17.07	1.214 (0.926, 1.592)		1.032 (0.783, 1.360)	
	**Pre-DM**	1587	15.94	1.151 (0.936, 1.415)		1.255 (1.012, 1.556)	
**Female**	**Non-DM**	2197	10.46	1 (Ref.)		1 (Ref.)	
	**Early PTDM**	398	13.06	1.247 (0.855, 1.819)		1.064 (0.726, 1.559)	
	**Pre-DM**	829	14.90	1.443 (1.087, 1.916)		1.350 (1.011, 1.803)	
**Income, Low**					0.6687		0.1510
**No**	**Non-DM**	3655	11.71	1 (Ref.)		1 (Ref.)	
	**Early PTDM**	714	13.95	1.194 (0.91, 1.567)		1.084 (0.822, 1.430)	
	**Pre-DM**	1683	13.73	1.192 (0.97, 1.467)		1.161 (0.937, 1.438)	
**Yes**	**Non-DM**	1383	14.72	1 (Ref.)		1 (Ref.)	
	**Early PTDM**	318	19.40	1.313 (0.902, 1.910)		0.986 (0.674, 1.442)	
	**Pre-DM**	733	20.21	1.396 (1.051, 1.854)		1.583 (1.184, 2.116)	
**Smoking status**					0.1267		0.0896
**Never**	**Non-DM**	3227	11.20	1 (Ref.)		1 (Ref.)	
	**Early PTDM**	627	13.62	1.216 (0.905, 1.636)		1.044 (0.773, 1.412)	
	**Pre-DM**	1354	14.98	1.358 (1.086, 1.697)		1.294 (1.029, 1.628)	
**Ex**	**Non-DM**	1512	13.37	1 (Ref.)		1 (Ref.)	
	**Early PTDM**	337	18.89	1.420 (0.989, 2.039)		1.270 (0.880, 1.832)	
	**Pre-DM**	882	13.10	0.998 (0.739, 1.348)		1.140 (0.839, 1.547)	
**Current**	**Non-DM**	299	22.84	1 (Ref.)		1 (Ref.)	
	**Early PTDM**	68	16.76	0.726 (0.326, 1.619)		0.542 (0.242, 1.212)	
	**Pre-DM**	180	33.78	1.500 (0.941, 2.392)		1.771 (1.100, 2.850)	
**Alcohol consumption**					0.3635		0.1859
**None**	**Non-DM**	4277	11.89	1 (Ref.)		1 (Ref.)	
	**Early PTDM**	888	15.98	1.345 (1.063, 1.702)		1.169 (0.919, 1.488)	
	**Pre-DM**	2126	14.85	1.269 (1.056, 1.524)		1.276 (1.053, 1.544)	
**Moderate**	**Non-DM**	725	15.74	1 (Ref.)		1 (Ref.)	
	**Early PTDM**	137	11.89	0.752 (0.388, 1.456)		0.522 (0.268, 1.016)	
	**Pre-DM**	265	22.04	1.411 (0.935, 2.129)		1.401 (0.923, 2.128)	
**Heavy**	**Non-DM**	36	18.97	1 (Ref.)		1 (Ref.)	
	**Early PTDM**	7	22.43	1.182 (0.132, 10.574)		1.603 (0.178, 14.474)	
	**Pre-DM**	25	7.64	0.419 (0.047, 3.747)		0.813 (0.091, 7.287)	
**Regular exercise**					0.4803		0.6307
**No**	**Non-DM**	3837	13.26	1 (Ref.)		1 (Ref.)	
	**Early PTDM**	762	17.22	1.299 (1.018, 1.657)		1.050 (0.818, 1.348)	
	**Pre-DM**	1759	16.08	1.232 (1.018, 1.490)		1.235 (1.012, 1.507)	
**Yes**	**Non-DM**	1201	10.06	1 (Ref.)		1 (Ref.)	

	**Early PTDM**	270	10.66	1.060 (0.635, 1.770)		1.017 (0.608, 1.703)	
	**Pre-DM**	657	14.23	1.437 (1.013, 2.037)		1.480 (1.039, 2.109)	
**Hypertension**					0.0853		0.1513
**No**	**Non-DM**	1835	8.26	1 (Ref.)		1 (Ref.)	
	**Early PTDM**	289	11.79	1.424 (0.898, 2.259)		1.196 (0.749, 1.910)	
	**Pre-DM**	711	13.73	1.692 (1.220, 2.348)		1.709 (1.225, 2.385)	
**Yes**	**Non-DM**	3203	15.03	1 (Ref.)		1 (Ref.)	
	**Early PTDM**	743	17.01	1.133 (0.882, 1.455)		0.999 (0.774, 1.289)	
	**Pre-DM**	1705	16.36	1.105 (0.910, 1.341)		1.173 (0.959, 1.435)	
**Dyslipidemia**					0.5972		0.1213
**No**	**Non-DM**	2271	13.02	1 (Ref.)		1 (Ref.)	
	**Early PTDM**	365	13.95	1.072 (0.741, 1.551)		0.813 (0.559, 1.184)	
	**Pre-DM**	970	16.04	1.247 (0.971, 1.602)		1.116 (0.863, 1.443)	
**Yes**	**Non-DM**	2767	12.05	1 (Ref.)		1 (Ref.)	
	**Early PTDM**	667	16.38	1.360 (1.032, 1.794)		1.224 (0.925, 1.620)	
	**Pre-DM**	1446	15.24	1.287 (1.029, 1.610)		1.449 (1.149, 1.827)	
**Depression**					0.9037		0.9229
**No**	**Non-DM**	4798	12.43	1 (Ref.)		1 (Ref.)	
	**Early PTDM**	961	15.21	1.224 (0.975, 1.536)		1.050 (0.831, 1.326)	
	**Pre-DM**	2223	15.29	1.251 (1.051, 1.488)		1.278 (1.064, 1.533)	
**Yes**	**Non-DM**	240	13.94	1 (Ref.)		1 (Ref.)	
	**Early PTDM**	71	20.41	1.480 (0.626, 3.500)		0.985 (0.415, 2.334)	
	**Pre-DM**	193	19.08	1.383 (0.738, 2.591)		1.413(0.752, 2.655)	
**Proteinuria**					0.1707		0.1653
**Negative**	**Non-DM**	4567	9.18	1 (Ref.)		1 (Ref.)	
	**Early PTDM**	906	11.06	1.206 (0.919, 1.583)		1.179 (0.895, 1.554)	
	**Pre-DM**	2155	12.21	1.360 (1.114, 1.659)		1.404 (1.142, 1.726)	
**Positive**	**Non-DM**	471	53.35	1 (Ref.)		1 (Ref.)	
	**Early PTDM**	126	57.93	1.085 (0.747, 1.576)		0.831 (0.569, 1.215)	
	**Pre-DM**	261	50.64	0.958 (0.705, 1.301)		1.050 (0.767, 1.438)	
**Hospitalization within one year post-KT**					0.5232		0.3073
**No**	**Non-DM**	1344	13.06	1 (Ref.)		1 (Ref.)	
	**Early PTDM**	181	14.31	1.095 (0.668, 1.795)		1.139 (0.692, 1.877)	
	**Pre-DM**	398	13.33	1.045 (0.708, 1.541)		1.005 (0.678, 1.488)	
**Yes**	**Non-DM**	3694	12.26	1 (Ref.)		1 (Ref.)	
	**Early PTDM**	851	15.77	1.285 (1.003, 1.645)		1.042 (0.809, 1.342)	
	**Pre-DM**	2018	16.06	1.324 (1.097, 1.598)		1.374 (1.129, 1.671)	
**Acute rejection therapy withing one year post-KT**					0.3498		0.3014
**No**	**Non-DM**	4727	11.64	1 (Ref.)		1 (Ref.)	
	**Early PTDM**	920	14.67	1.262 (0.996, 1.599)		1.101 (0.865, 1.403)	
	**Pre-DM**	2216	14.79	1.292 (1.081, 1.544)		1.343 (1.114, 1.620)	
**Yes**	**Non-DM**	311	26.63	1 (Ref.)		1 (Ref.)	
	**Early PTDM**	112	23.37	0.874 (0.483, 1.584)		0.749 (0.412, 1.360)	
	**Pre-DM**	200	25.00	0.949 (0.590, 1.528)		0.961 (0.596, 1.551)	

Adjusted HR was adjusted for sex, age, low income, smoking status, alcohol consumption, regular exercise, hypertension, dyslipidemia, depression, BMI, GFR, time from KT to screening, proteinuria, induction therapy, and acute rejection within one year

CI, confidence interval; DM, diabetes insipidus; HR, hazard ratio; IR, incidence rate; KT, kidney transplantation; PTDM, post-transplantion diabetes mellitus

Supplementary Table S3. Subgroup analysis of all-cause mortality according to diabetic status within one year post-KT

Subgroup	diabetic status	N	IR	Unadjusted HR (95% CI)	P for interaction	Adjusted HR (95% CI)	P for interaction
**Age group (years)**					0.7201		0.7413
**<40**	**Non-DM**	1022	2.46	1 (Ref.)		1(Ref.)	
	**Early PTDM**	75	5.34	2.131 (0.628, 7.235)		1.674 (0.492, 5.700)	
	**Pre-DM**	166	2.85	1.220 (0.359, 4.143)		1.062 (0.312, 3.616)	
**40–49**	**Non-DM**	1693	3.92	1 (Ref.)		1 (Ref.)	
	**Early PTDM**	251	3.44	0.873 (0.372, 2.046)		0.736 (0.313, 1.727)	
	**Pre-DM**	562	9.33	2.492 (1.583, 3.922)		2.328 (1.475, 3.672)	
**50–59**	**Non-DM**	1646	6.81	1 (Ref.)		1 (Ref.)	
	**Early PTDM**	449	12.65	1.852 (1.253, 2.736)		1.622 (1.094, 2.404)	
	**Pre-DM**	985	15.14	2.302 (1.696, 3.126)		2.150 (1.578, 2.929)	
**60–69**	**Non-DM**	631	17.81	1 (Ref.)		1 (Ref.)	
	**Early PTDM**	230	24.21	1.360 (0.896, 2.063)		1.250 (0.822, 1.903)	
	**Pre-DM**	623	36.59	2.149 (1.595, 2.895)		2.035 (1.505, 2.751)	
**≥70**	**Non-DM**	46	38.56	1 (Ref.)		1 (Ref.)	
	**Early PTDM**	27	42.87	1.102 (0.392, 3.094)		1.136 (0.402, 3.213)	
	**Pre-DM**	80	71.18	1.898 (0.886, 4.065)		1.848 (0.857, 3.983)	
**Sex**					0.0696		0.3419
**Male**	**Non-DM**	2841	6.65	1 (Ref.)		1 (Ref.)	
	**Early PTDM**	634	13.53	2.052 (1.499, 2.811)		1.324 (0.963, 1.821)	
	**Pre-DM**	1587	22.67	3.558 (2.852, 4.439)		2.255 (1.795, 2.832)	
**Female**	**Non-DM**	2197	5.91	1 (Ref.)		1 (Ref.)	
	**Early PTDM**	398	11.22	1.894 (1.251, 2.869)		1.308 (0.862, 1.985)	
	**Pre-DM**	829	13.03	2.280 (1.653, 3.144)		1.710 (1.234, 2.369)	
**Income, Low**					0.7224		0.8128
**No**	**Non-DM**	3655	6.14	1 (Ref.)		1 (Ref.)	
	**Early PTDM**	714	11.78	1.926 (1.421, 2.610)		1.239 (0.911, 1.685)	
	**Pre-DM**	1683	19.26	3.251 (2.63, 4.020)		2.037 (1.636, 2.537)	
**Yes**	**Non-DM**	1383	6.84	1 (Ref.)		1 (Ref.)	
	**Early PTDM**	318	14.67	2.151 (1.382, 3.347)		1.478 (0.946, 2.310)	
	**Pre-DM**	733	19.36	2.959 (2.118, 4.135)		2.132 (1.519, 2.993)	
**Smoking status**					0.0771		0.2591
**Never**	**Non-DM**	3227	6.22	1 (Ref.)		1 (Ref.)	
	**Early PTDM**	627	12.86	2.070 (1.510, 2.838)		1.346 (0.979, 1.850)	
	**Pre-DM**	1354	16.51	2.751 (2.165, 3.495)		1.846 (1.445, 2.359)	
**Ex**	**Non-DM**	1512	6.50	1 (Ref.)		1 (Ref.)	
	**Early PTDM**	337	11.99	1.874 (1.190, 2.950)		1.249 (0.790, 1.977)	
	**Pre-DM**	882	19.49	3.133 (2.293, 4.280)		2.130 (1.551, 2.924)	
**Current**	**Non-DM**	299	6.57	1 (Ref.)		1(Ref.)	
	**Early PTDM**	68	13.42	1.991 (0.757, 5.240)		1.360 (0.515, 3.591)	
	**Pre-DM**	180	40.61	6.437 (3.449, 12.015)		3.661 (1.946, 6.886)	
**Alcohol consumption**					0.9828		0.9441
**None**	**Non-DM**	4277	6.24	1 (Ref.)		1 (Ref.)	
	**Early PTDM**	888	12.42	1.996 (1.520, 2.620)		1.320 (1.002, 1.739)	
	**Pre-DM**	2126	19.31	3.219 (2.651, 3.908)		2.105 (1.721, 2.574)	
**Moderate**	**Non-DM**	725	6.72	1 (Ref.)		1 (Ref.)	
	**Early PTDM**	137	13.56	2.041 (1.054, 3.953)		1.195 (0.612, 2.334)	
	**Pre-DM**	265	18.19	2.788 (1.693, 4.592)		1.742 (1.052, 2.884)	
**Heavy**	**Non-DM**	36	8.46	1 (Ref.)		1 (Ref.)	
	**Early PTDM**	7	20.08	2.262 (0.205, 24.955)		2.155 (0.193, 24.089)	
	**Pre-DM**	25	29.85	3.808 (0.697, 20.797)		3.158 (0.572, 17.428)	
**Regular exercise**					0.5877		0.3636
**No**	**Non-DM**	3837	6.57	1 (Ref.)		1 (Ref.)	
	**Early PTDM**	762	12.70	1.936 (1.452, 2.581)		1.246 (0.931, 1.668)	
	**Pre-DM**	1759	20.77	3.285 (2.683, 4.021)		2.152 (1.745, 2.655)	
**Yes**	**Non-DM**	1201	5.56	1 (Ref.)		1 (Ref.)	

	**Early PTDM**	270	12.40	2.259 (1.357, 3.762)		1.530 (0.916, 2.556)	
	**Pre-DM**	657	15.38	2.882 (1.957, 4.243)		1.783 (1.206, 2.637)	
**Hypertension**					0.3478		0.5623
**No**	**Non-DM**	1835	5.70	1 (Ref.)		1 (Ref.)	
	**Early PTDM**	289	14.25	2.488 (1.606, 3.853)		1.567 (1.008, 2.435)	
	**Pre-DM**	711	16.46	2.992 (2.15, 4.164)		2.029 (1.451, 2.838)	
**Yes**	**Non-DM**	3203	6.69	1 (Ref.)		1 (Ref.)	
	**Early PTDM**	743	11.96	1.801 (1.328, 2.444)		1.210 (0.889, 1.648)	
	**Pre-DM**	1705	20.50	3.188 (2.573, 3.950)		2.073 (1.661, 2.587)	
**Dyslipidemia**					0.9004		0.7819
**No**	**Non-DM**	2271	6.32	1 (Ref.)		1 (Ref.)	
	**Early PTDM**	365	13.66	2.154 (1.458, 3.182)		1.206 (0.812, 1.792)	
	**Pre-DM**	970	19.86	3.236 (2.474, 4.234)		1.936 (1.471, 2.550)	
**Yes**	**Non-DM**	2767	6.33	1 (Ref.)		1 (Ref.)	
	**Early PTDM**	667	12.02	1.913 (1.379, 2.653)		1.391 (1.001, 1.933)	
	**Pre-DM**	1446	18.88	3.118 (2.451, 3.966)		2.172 (1.697, 2.781)	
**Depression**					0.8306		0.843
**No**	**Non-DM**	4798	6.10	1 (Ref.)		1 (Ref.)	
	**Early PTDM**	961	11.97	1.968 (1.512, 2.561)		1.281 (0.980, 1.673)	
	**Pre-DM**	2223	18.40	3.141 (2.601, 3.794)		2.060 (1.693, 2.508)	
**Yes**	**Non-DM**	240	11.29	1 (Ref.)		1 (Ref.)	
	**Early PTDM**	71	23.59	2.167 (0.966, 4.862)		1.634 (0.721, 3.701)	
	**Pre-DM**	193	30.29	2.771 (1.539, 4.991)		2.127 (1.176, 3.845)	
**Proteinuria**					0.2480		0.4375
**Negative**	**Non-DM**	4567	5.77	1 (Ref.)		1 (Ref.)	
	**Early PTDM**	906	10.08	1.755 (1.312, 2.347)		1.208 (0.900, 1.620)	
	**Pre-DM**	2155	17.38	3.136 (2.573, 3.822)		2.076 (1.691, 2.549)	
**Positive**	**Non-DM**	471	12.10	1 (Ref.)		1 (Ref.)	
	**Early PTDM**	126	32.81	2.710 (1.627, 4.514)		1.684 (1.002, 2.830)	
	**Pre-DM**	261	36.31	3.094 (2.022, 4.733)		2.035 (1.322, 3.132)	
**Hospitalization within one year post-KT**					0.5350		0.3330
**No**	**Non-DM**	1344	5.27	1(Ref.)		1 (Ref.)	
	**Early PTDM**	181	13.50	2.577(1.510, 4.399)		1.832 (1.071, 3.133)	
	**Pre-DM**	398	16.84	3.367(2.245, 5.051)		2.058 (1.365, 3.104)	
**Yes**	**Non-DM**	3694	6.77	1(Ref.)		1 (Ref.)	
	**Early PTDM**	851	12.41	1.827(1.376, 2.425)		1.179 (0.885, 1.572)	
	**Pre-DM**	2018	19.81	3.012(2.463, 3.685)		2.010 (1.632, 2.474)	
**Acute rejection therapy withing one year post-KT**					0.0265		0.0704
**No**	**Non-DM**	4727	6.39	1 (Ref.)		1 (Ref.)	
	**Early PTDM**	920	11.07	1.738 (1.321, 2.287)		1.174 (0.889, 1.548)	
	**Pre-DM**	2216	18.82	3.062 (2.541, 3.690)		1.999 (1.645, 2.429)	
**Yes**	**Non-DM**	311	5.33	1 (Ref.)		1 (Ref.)	
	**Early PTDM**	112	26.79	5.070 (2.395, 10.734)		3.004 (1.412, 6.389)	
	**Pre-DM**	200	24.62	4.791 (2.385, 9.624)		3.289 (1.635, 6.618)	

Adjusted HR was adjusted for sex, age, low income, smoking status, alcohol consumption, regular exercise, hypertension, dyslipidemia, depression, BMI, GFR, time from KT to screening, proteinuria, induction therapy, and acute rejection within one year

CI, confidence interval; DM, diabetes insipidus; HR, hazard ratio; IR, incidence rate; KT, kidney transplantation; PTDM, post-transplantion diabetes mellitus

Supplementary Table S4. Risk of graft failure and mortality according to diabetic status within one year post-KT: early PTDM defined as onset within the first year after KT

	Diabetic status	IR[a]	Hazard ratio (95% confidence interval)			
			**Model 1**	**Model 2**	**Model 3**	**Model 4**
**Graft failure**	**Non-DM**	12.78	1 (Ref.)	1 (Ref.)	1 (Ref.)	1 (Ref.)
	**Early PTDM**	13.42	1.048 (0.870, 1.262)	1.114 (0.923 ,1.344)	1.079 (0.894, 1.302)	0.903 (0.746, 1.093)
	**Preexisting DM**	15.57	1.236 (1.040, 1.469)	1.365 (1.140 ,1.634)	1.310 (1.094, 1.569)	1.227 (1.022, 1.472)
**All-cause mortality**	**Non-DM**	6.60	1 (Ref.)	1 (Ref.)	1 (Ref.)	1 (Ref.)
	**Early PTDM**	9.04	1.370 (1.086, 1.727)	1.083 (0.858, 1.367)	1.071 (0.848, 1.351)	0.996 (0.788, 1.259)
	**Preexisting DM**	19.29	3.036 (2.515, 3.664)	1.937 (1.597, 2.350)	1.874 (1.543, 2.275)	1.913 (1.574, 2.326)

Model 1 was unadjusted; Model 2 was adjusted for sex and age; Model 3 was further adjusted for low income, smoking status, alcohol consumption, and regular exercise; and Model 4 was additionally adjusted for hypertension, dyslipidemia, depression, body mass index, estimated glomerular filtration rate, proteinuria, time from kidney transplantation to health screening, induction therapy, and acute rejection within one year.

[a]Incidence rates are expressed as per 1000 person-year

DM, diabetes mellitus; IR, incidence rate; KT, kidney transplantation; PTDM, post-transplantion diabetes mellitus

Supplementary Table S5. Risk of graft failure and mortality according to the changes in diabetic status: early PTDM defined as onset within the first year after KT

	Changes in diabetic status		N	IR[a]	Hazard ratio (95% confidence interval)			
	**With 1 year after KT**	**At health screening**			**Model 1**	**Model 2**	**Model 3**	**Model 4**
**Graft failure**	**Non-DM**	**Non-DM**	3,154	12.39	1 (Ref.)	1 (Ref.)	1 (Ref.)	1 (Ref.)
	**Non-DM**	**PTDM**	230	23.23	1.883 (1.297, 2.736)	1.956 (1.345, 2.844)	1.783 (1.221, 2.606)	1.578 (1.074, 2.320)
	**Early PTDM**	**Non-DM**	864	13.62	1.094 (0.848, 1.413)	1.158 (0.896, 1.496)	1.144 (0.885, 1.479)	0.955 (0.734, 1.241)
	**Early PTDM**	**PTDM**	735	14.12	1.140 (0.866, 1.500)	1.309 (0.987, 1.734)	1.198 (0.902, 1.591)	0.983 (0.736, 1.313)
**All-cause mortality**	**Non-DM**	**Non-DM**	3,154	6.31	1 (Ref.)	1 (Ref.)	1 (Ref.)	1 (Ref.)
	**Non-DM**	**PTDM**	230	13.84	2.208 (1.38, 3.532)	2.042 (1.276, 3.268)	2.067 (1.284, 3.326)	1.784 (1.100, 2.893)
	**Early PTDM**	**Non-DM**	864	5.71	0.899 (0.617, 1.310)	0.783 (0.537, 1.142)	0.769 (0.527, 1.122)	0.678 (0.464, 0.992)
	**Early PTDM**	**PTDM**	735	15.24	2.421 (1.820, 3.219)	1.699 (1.273, 2.269)	1.693 (1.265, 2.265)	1.562 (1.162, 2.101)

Model 1 was unadjusted; Model 2 was adjusted for sex and age; Model 3 was further adjusted for low income, smoking status, alcohol consumption, and regular exercise; and Model 4 was additionally adjusted for hypertension, dyslipidemia, depression, body mass index, estimated glomerular filtration rate, proteinuria, time from kidney transplantation to health screening, induction therapy, and acute rejection within one year.

[a]Incidence rates are expressed as per 1000 person-year

DM, diabetes mellitus; IR, incidence rate; KT, kidney transplantation; PTDM, post-transplantion diabetes mellitus

Supplementary Table S6. Baseline characteristics of all KT recipients who received KT from 2004 to 2020 regardless of national health screening program participation.

	All (N = 25,242)	Non-DM (n = 13,546)	Early PTDM (n = 2,742)	Preexisting DM (n = 8,954)	P- value
**Age, y**	46.9 ± 12.59	42.97 ± 12.87	50.57 ± 11.04	51.73 ± 10.38	< 0.0001
**<40**	6,695 (26.52)	5,078 (37.49)	428 (15.61)	1,189 (13.28)	< 0.0001
**40–49**	6,768 (26.81)	3,952 (29.17)	676 (24.65)	2,140 (23.9)	
**50–59**	7,876 (31.20)	3,306 (24.41)	1,066 (38.88)	3,504 (39.13)	
**60–69**	3,578 (14.17)	1,119 (8.26)	526 (19.18)	1,933 (21.59)	
**≥70**	325 (1.29)	91 (0.67)	46 (1.68)	188 (2.10)	
**Male sex**	15,066 (59.69)	7,510 (55.44)	1,625 (59.26)	5,931 (66.24)	< 0.0001
**Hypertension**	23,232 (92.04)	1,2196 (90.03)	2,497 (91.06)	8,539 (95.37)	< 0.0001
**Dyslipidemia**	11,913 (47.20)	5,203 (38.41)	1,220 (44.49)	5,490 (61.31)	< 0.0001
**Low income (<20%)**	7,283 (28.85)	3,865 (28.53)	773 (28.19)	2,645 (29.54)	0.190
**Induction therapy**					< 0.0001
**None**	1,828 (7.24)	1,291 (9.53)	192 (7.00)	345 (3.85)	
**Thymoglobulin**	3,410 (13.51)	1,605 (11.85)	325 (11.85)	1,480 (16.53)	
**Basiliximab**	19,232 (76.19)	10,259 (75.73)	2,133 (77.79)	6,840 (76.39)	
**Both**	772 (3.06)	391 (2.89)	92 (3.36)	289 (3.23)	
**Acute rejection within 1 year**	2,698 (10.69)	1,170 (8.64)	435 (15.86)	1,093 (12.21)	< 0.0001
**OPD visit within 1 year, n**	27.44 ± 16.19	24.95 ± 14.51	27.14 ± 14.44	31.29 ± 18.23	< 0.0001
**<10**	287 (1.14)	160 (1.18)	28 (1.02)	99 (1.11)	< 0.0001
**10–19**	7,950 (31.5)	5,280 (38.98)	846 (30.85)	1,824 (20.37)	
**20–29**	9,092 (36.02)	4,845 (35.77)	1,007 (36.73)	3,240 (36.18)	
**30–39**	4,379 (17.35)	1,929 (14.24)	484 (17.65)	1,966 (21.96)	
**≥40**	3,534 (14.00)	1,332 (9.83)	377 (13.75)	1,825 (20.38)	
**Admission within 1 year, n**	2.05 ± 2.07	1.74 ± 1.76	2.37 ± 2.28	2.44 ± 2.33	< 0.0001
**0**	4,346 (17.22)	2,829 (20.88)	379 (13.82)	1,138 (12.71)	< 0.0001
**1**	8,017 (31.76)	4,654 (34.36)	790 (28.81)	2,573 (28.74)	
**≥2**	12,879 (51.02)	6,063 (44.76)	1,573 (57.37)	5,243 (58.55)	

Categorical variables and continuous variables are presented as number (percentage) and as mean ± standard deviation.

DM, diabetes mellitus; KT, kidney transplantation; PTDM, post-transplantion diabetes mellitus

Supplementary Table S7. Risk of graft failure and mortality by diabetic status within 1 year KT in all KTRs who received KT from 2004 to 2020 regardless of health screening participation.

	Diabetic status	IR[a]	Hazard ratio (95% confidence interval)		
			Model 1	Model 2	Model 3
**Graft failure**	**Non-DM**	11.39	1 (Ref.)	1 (Ref.)	1 (Ref.)
	**Early PTDM**	17.32	1.525 (1.349, 1.725)	1.708 (1.506, 1.936)	1.524 (1.344, 1.729)
	**Preexisting DM**	15.24	1.385 (1.264, 1.519)	1.579 (1.433, 1.741)	1.462 (1.324, 1.614)
**All-cause mortality**	**Non-DM**	6.22	1 (Ref.)	1 (Ref.)	1 (Ref.)
	**Early PTDM**	13.24	2.142 (1.861, 2.465)	1.474 (1.279, 1.700)	1.330 (1.153, 1.535)
	**Preexisting DM**	20.03	3.432 (3.113, 3.783)	2.161 (1.952, 2.392)	2.108 (1.900, 2.338)

Model 1: unadjusted

Model 2: sex and age

Model 3: sex, age, low income, hypertension, dyslipidemia, induction therapy, and acute rejection within one year

[a]Incidence rates are expressed as per 1000 person-year

DM, diabetes insipidus; IR, incidence rate; KT, kidney transplantation; PTDM, post-transplantion diabetes mellitus
